# Determinants of Drug Resistance in B-Cell Non-Hodgkin Lymphomas: The Case of Lymphoplasmacytic Lymphoma/Waldenström Macroglobulinemia

**DOI:** 10.3389/fonc.2021.801124

**Published:** 2022-01-11

**Authors:** Francesco Piazza, Veronica Di Paolo, Greta Scapinello, Sabrina Manni, Livio Trentin, Luigi Quintieri

**Affiliations:** ^1^ Laboratory of Myeloma and Lymphoma Pathobiology, Veneto Institute of Molecular Medicine (VIMM) and Foundation for Advanced Biomedical Research (FABR), Padua, Italy; ^2^ Hematology Division, Azienda Ospedaliera Universitaria and Department of Medicine, University of Padua, Padua, Italy; ^3^ Laboratory of Drug Metabolism, Department of Pharmaceutical and Pharmacological Sciences, University of Padua, Padua, Italy

**Keywords:** lymphoplasmacytic lymphoma, Waldenström macroglobulinaemia, drug resistance, MyD88, CXCR4, Bruton’s tyrosine kinase, BTK inhibitors, anti-CD20 monoclonal antibodies

## Abstract

Lymphoplasmacytic lymphoma (LPL) is a rare subtype of B cell-derived non-Hodgkin lymphoma characterized by the abnormal growth of transformed clonal lymphoplasmacytes and plasma cells. This tumor almost always displays the capability of secreting large amounts of monoclonal immunoglobulins (Ig) of the M class (Waldenström Macroglobulinemia, WM). The clinical manifestations of WM/LPL may range from an asymptomatic condition to a lymphoma-type disease or may be dominated by IgM paraprotein-related symptoms. Despite the substantial progresses achieved over the last years in the therapy of LPL/WM, this lymphoma is still almost invariably incurable and exhibits a propensity towards development of refractoriness to therapy. Patients who have progressive disease are often of difficult clinical management and novel effective treatments are eagerly awaited. In this review, we will describe the essential clinical and pathobiological features of LPL/WM. We will also analyze some key aspects about the current knowledge on the mechanisms of drug resistance in this disease, by concisely focusing on conventional drugs, monoclonal antibodies and novel agents, chiefly Bruton’s Tyrosine Kinase (BTK) inhibitors. The implications of molecular lesions as predictors of response or as a warning for the development of therapy resistance will be highlighted.

## Introduction

Lymphoplasmacytic Lymphoma (LPL) is a tumor involving mature small B lymphocytes, plasmacytoid lymphocytes and plasma cells. The growth of neoplastic cells usually involves the bone marrow and sometimes the lymph-nodes and spleen. LPL should be differentiated from other mature B cell lymphomas, especially Marginal Zone Lymphoma (1). The morphological features of LPL are characterized by the infiltrate in the involved organs of small B lymphocytes with aspects of plasma cellular differentiation as well as of a certain amount of plasma cells. When a paraprotein of the IgM class is produced by the neoplastic clone, the criteria for Waldenström Macroglobulinemia (WM) are fulfilled, a condition first described by the Swedish physician Jan Gösta Waldenström (2).

When symptomatic, the disease could produce systemic manifestations such as fatigue, weight loss, weakness and night sweats. Anemia is a frequent finding and is caused by the chronic inflammation associated with the disease (3) or by bone marrow (BM) infiltration by neoplastic cells. However, it is always mandatory to rule out other causes. Less frequently, if the BM is massively substituted, leukocytopenia and thrombocytopenia can also occur.

In WM, other clinical manifestations are related to the IgM paraprotein. When the monoclonal immunoglobulin reaches high serum concentrations it may cause serum hyperviscosity – due to its chemico-physical features of forming pentamers or hexamers. Clinically, patients may present with an array of symptoms, such as bleeding, blurred vision, hearing loss, vertigo, paresthesias, seizures, stupor, coma. The monoclonal IgM may also display autoimmune properties, usually directed against glycoproteins of the myelin of the peripheral nerves or against the erythrocytes, with the development of peripheral neuropathy and autoimmune hemolytic anemia, respectively. Amyloidosis due to the deposit of IgM paraprotein light chains can also occur in 5-10% of patients and is characterized by a frequent renal involvement (58%), cardiac infiltration (41%), neuropathy (23%) and lymph nodal substitution with amyloid (22%) (4).

The Bing-Neel syndrome is a rare manifestation of LPL/WM caused by the infiltration of the central nervous system by the neoplastic cells (5). Patients may present with a wide array of neurological signs and symptoms, depending on the site of involvement in the CNS. The onset is gradual over weeks or months. Seizures, headache, visual loss, cranial neuropathies, focal sensory or motor dysfunctions are common manifestations (5).

The molecular pathogenesis of LPL/WM is still largely unexplored. However, in the last years significant advancements have been achieved mostly thanks to the new methodologies available to explore the cancer genome, such as next generation sequencing (NGS), which has allowed the recognition of the *myeloid differentiation factor 88* (*MYD88*) L265P mutation as the most frequent molecular lesion associated to this disease (found in than 90% of cases) (6). Even if the *MYD88* L265P mutation is not pathognomonic for LPL/WM, its detection may support the diagnosis and has also a predictive value (6, 7). Other commonly recurring mutations involve the *C-X-C Motif Chemokine Receptor 4* **(** *CXCR4*; 30%; see below), *AT-Rich Interaction Domain 1A* (*ARID1A*; 17%), *Tumor Protein 53* (TP53; 8%), and *B-cell antigen receptor signaling component Igβ* (CD79B; 8% to 15%) genes (8).

## Molecular Pathogenesis and Mechanisms of Cell Growth in LPL/WM

The *MYD88* gene codes for a scaffold protein that in lymphoid cells mediates the signal downstream from the Interleukin-1, -6 and -8 and the Toll-like receptors (TLR) (9, 10). MYD88 protein has at its N-terminus a death domain (DD), in the center an intermediate linker domain (ILD) and at its C-terminus a Toll/IL-1R domain (TIR). MYD88 associates in a TIR-mediated interaction with the intracellular portion of the receptors and with the cytoplasmic serine-threonine kinase IL-1R activated kinase 4 (IRAK4) through the DD, which in turn associates with and phosphorylates the kinases IRAK1 and IRAK2 in the so called “Myddosome” (11). The E3-ubiquitin ligase Tumor Necrosis Factor Receptor associated factor 6 (TRAF6) is then recruited and in turn binds to TAK1-binding protein 2 (TAB2) and activates the TGFβ activated kinase 1 (TAK1), thereby triggering the downstream activation of the nuclear factor of activated B cells (NF-κB) transcription factor and mitogen activated kinase (MAPK) signaling pathways (12, 13). LPL/WM cells harboring the *MYD88* L265P mutation in the TIR domain display a constitutively active NF-κB pathway. It has been demonstrated that the small proportion of *MYD88* wild-type LPL patients tend to display a lower bone marrow and splenic involvement and a worse clinical course (6). Other rarer mutations to *MYD88* have also been described whose precise biological and clinical significance is still under evaluation (14).

The *CXCR4* gene presents mutations in the C-terminal domain similar to those seen in the WHIM (Warts, Hypogammaglobulinemia, Infections and Myelokathexis) congenital immunodeficiency syndrome (6, 15). The most common (>50% of cases) is the *CXCR4* S338X (C1013G) mutation, which causes a truncation of the C-terminal regulatory region of the protein. Overall, LPL/WM patients with the CXCR4 mutations are approximately 30% of all cases. CXCR4 is involved in cell recycling and migration to the bone marrow protective niche (16). It has been demonstrated that the CXCR4 S338X mutation confers a hyperactive state to the receptor, leading to a downstream constitutive activation of the ERK and AKT survival signaling pathways and resistance to the cytotoxic effects of several drugs, including Bruton’s tyrosine kinase (BTK), phosphoinositide 3-kinases (PI3K), B*-*cell leukemia*/*lymphoma*-*2 (BCL2) and proteasome inhibitors (17).

## Prognostication and Therapy of LPL/WM

Symptomatic patients in need of therapy are stratified according to the International Waldenström Macroglobulinemia Scoring System (IWMSS), which recognizes three risk categories (low, intermediate and high risk) according to the presence of 0-1, 2 or >2 of five adverse covariates (age >65 years, hemoglobin ≤11.5 g/dL, platelets ≤100 x 10^9/L, beta2-microglobulin >3mg/L, serum monoclonal protein >7.0 g/dL), respectively (18).

According to several guidelines, first line therapy may be dictated by the molecular profile of the disease and by the tumor burden and/or critical involvement of target organs. In the most frequent scenario of *MYD88^mut^ CXCR4^wt^* cases with no need of rapid debulking, rational options are BTK inhibitors plus rituximab or chemoimmunotherapy with the association of rituximab, a monoclonal anti-CD20 antibody and an alkylating agent, such as bendamustine or cyclophosphamide. When a rapid debulking is required to alleviate symptoms caused by hyperviscosity or avoid an incipient kidney damage, chemoimmunotherapy or proteasome inhibitors, i.e. bortezomib, are advised (19). These treatments generally induce durable responses, are typically well-tolerated and remain essential in the management of the disease (7, 20). However, LPL/WM almost invariably relapses, and the goal of therapy is the control of symptoms and the achievement of the deepest and longest response. Subsequent therapies should either re-utilize effective first-line regimens or make use of non-cross resistant drugs. Depending on whether the patient had been exposed to alkylating agents or nucleoside analogs in the first-line therapy, the second-line therapy will use either one of the class of drugs. Along this line, novel treatment choices are necessary for patients who become rituximab-refractory or intolerant (21). In this regard, inhibition of BTK, a downstream cytoplasmic kinase in the MYD88 signaling pathway whose expression is limited to cells of the hematopoietic lineage, excluding T-cells, has increasingly gained attention as a strategy for the therapy of LPL/WM. Indeed, as stated above, the gain-of-function *MYD88* L265P mutation harbored by far more than 90% of WM patients, triggers the activation of BTK, leading to increased cell survival and proliferation through the NF-κB pathway (22). To note, it should be mentioned that the diagnostic methodologies of detection of *MYD88* and *CXCR4* mutations are still not universally standardized, with evidence of essential differences described in clinical trials (23, 24).

## Mechanisms of Drug Resistance in LPL/WM

### Chemotherapy

Conventional chemotherapy for LPL/WM is represented mostly by alkylating agents (chlorambucil, melphalan and cyclophosphamide) and nucleoside analogs [fludarabine, pentostatine, cladribine) as well as bendamustine (structurally containing both alkylating and nucleoside-analog moieties (25)] and is still a fundamental part of the treatment of this lymphoid malignancy (7, 20). As for most of the low-grade lymphomas, LPL/WM displays an initial sensitivity to chemotherapy, which fades out each time a new therapy is applied due to the relapse of the disease, prompting the use of different non-cross-resistant drugs. Eventually, the disease will be composed by predominant neoplastic cellular clones displaying a multidrug-resistant (MDR) phenotype (26, 27). The mechanisms of resistance are, in general, common to other types of malignant tumors, involving the overexpression of efflux drug transporters, mutations in TP53 and overexpression of oncogenic pathways, such as the mitogen-activated protein kinase (MAPK) and the PI3K/AKT cascades (28, 29). Despite several cytogenetic and molecular alterations have been recognized to possess a prognostic role in LPL/WM, their precise significance in the resistance to chemotherapeutic drugs is still unknown (30).

### Immunotherapy

As far as the anti-CD20 drugs are considered, in particular rituximab, resistance is conventionally defined as the lack of response to a rituximab-containing regimen, or progression within 6 months of treatment with a rituximab-containing regimen (31). Data have demonstrated that resistance mechanisms may involve the anti-CD20-dependent immune-mediated cytotoxic mechanisms, such as the antibody-dependent cellular cytotoxicity (ADCC), complement-dependent cytotoxicity (CDC) and antibody-dependent cellular phagocytosis (ADCP) (31). In some instances, it has been described that these effector mechanisms may be recovered by employing strategies aimed at reactivating the immune system. However, the clinical applicability of such approaches is still unfeasible (32–34). Caution should also be practiced, since complement activation, though important for rituximab activity, may play also a role in infusion reactions (35) and, consequently, further augmenting the activation of the complement cascade would lead to potentially deleterious consequences. Yet, the lower level of complement activation exerted by another anti-CD20 monoclonal antibody, obinutuzumab, for which infusion reactions are also well recognized, would suggest the implication of other mechanisms (36).

A likely mechanism of resistance to anti-CD20 therapies is represented by the aberrant cytosolic membrane dynamics of the CD20 molecules, which lies normally within membrane lipid rafts, structure rich of cholesterol and glycosphingolipids that have been found altered in a variety of B-cell non-Hodgkin lymphomas (NHL) and that could also be impaired by statins use (37, 38). Also, genetic mutations of the *CD20* gene have been described in NHL that cause a deletion of the antibody binding domain, thus causing an escape to anti-CD20 targeting (39).

The genetic polymorphism of the *FCGRA* gene coding for Fc gamma receptor (FcgR), the receptor that binds the Fc portion of immunoglobulins of G class, has also been associated to a different efficacy of monoclonal antibodies and potential resistance (31). Polymorphism of the FcgR affects the affinity of binding to the antibody and thus the ADCC. Patients bearing the single nucleotide polymorphism that substitutes a Valine (V) at residue 158 in homozygosity (158V/V) of the FcgIIIAR show a higher binding affinity as compared with individuals bearing a phenylalanine (F) substitution (158F/F) (40). Clinical data have reported worse outcomes for NHL patients with a 158F/F polymorphism (41).

### Proteasome Inhibitors

Targeting the ubiquitin proteasome pathway is another option in the therapy of LPL/WM and currently employed drugs are the first-in-class bortezomib, the second-generation carfilzomib and the newest agents ixazomib and oprozomib (20). Mechanisms of resistance to proteasome inhibitors in LPL/WM are poorly known. However, research in cell lines and in *in vivo* mouse models have demonstrated that proteasome inhibitor resistance may depend on rewiring of the proteasome machinery, by upregulating deubiquitinating enzymes (42), by BCL2 overexpression (42–44) or interaction with phorbol-12-myristate-13-acetate-induced protein 1 (PMAIP1; NOXA) (45) or by epigenetic deregulation of the TP63 and CCAAT/enhancer binding protein (CEBPA) signaling (46). Further research is needed to explore in depth the mechanisms of resistance to PIs in LPL/WM.

### BTK Inhibitors

BTK has been demonstrated to be an important therapeutic target in LPL/WM. Ibrutinib is an orally active, first-generation BTK inhibitor that covalently (i.e., irreversibly) binds to the cysteine 481 (C481) residue located in the ATP-binding pocket of the enzyme (47). Ibrutinib is currently approved, both alone and in combination with rituximab, by the US Food and Drug Administration (FDA) and the European Medicines Agency (EMA) for the treatment of several B-cell malignancies, including WM. Besides inhibiting BTK, ibrutinib also irreversibly inhibits various other kinases which contain a cysteine residue aligning with C481 in BTK, including but not limited to tyrosine kinase expressed in hepatocellular carcinoma (TEC), interleukin-2-inducible T-cell kinase (ITK), and epidermal growth factor receptor (EGFR) (48). Inhibition of off-target kinases is thought to underlie many of the adverse effects of this drug in the clinical setting, including diarrhea, rash, hypertension, atrial fibrillation, and platelet disfunction leading to bleeding complications (49). On the other hand, ibrutinib behaves also as a potent non-covalent inhibitor of several SRC family members, including hematopoietic cell kinase (HCK) (48, 50). Inhibition of HCK may contribute to the therapeutic efficacy of ibrutinib, since this kinase is overexpressed and hyperactive in *MYD88* L265P WM cells and supports tumor cell survival and growth (50).

Three clinical studies have evaluated the efficacy and safety of ibrutinib as a once-daily single-agent in patients with WM: two studies in patients with relapsed and/or refractory WM (51–53), and one in previously untreated patients (54). In these clinical trials, ibrutinib treatment was generally well tolerated and led to high rates of durable response. A fourth clinical study (iNNOVATE) evaluated the combination of ibrutinib and rituximab in WM treatment; the use of ibrutinib–rituximab resulted in a significantly higher rate of progression-free survival than the use of placebo–rituximab, both among patients who were treatment naïve (TN), and among those who had relapsed disease after response to a rituximab-containing regimen (55). Interestingly, no novel toxicities emerged, whereas the treatment discontinuation rate due to toxicity was similar between the two treatment groups. However, this study does not answer the question whether rituximab adds value to ibrutinib alone since it lacks an ibrutinib monotherapy arm. The updated long-term data of these pivotal studies of ibrutinib in the relapse and front-line setting and of the iNNOVATE trial have been recently published, further confirming the efficacy and safety profiles (56–58).

Clinical response to ibrutinib was found to be influenced by *MYD88* and *CXCR4* mutational status. No major responses to ibrutinib alone were recorded in previously treated WM patients who were wild-type *MYD88* (51, 59). Moreover, *MYD88* wild-type patients receiving ibrutinib monotherapy had much shorter progression free survival compared with *MYD88*-mutated patients, regardless of their *CXCR4* mutational status (60). In WM, primary resistance to ibrutinib is seen in patients with *CXCR4* WHIM mutations; patients harboring the *MYD88* L265P mutation who were wild-type for *CXCR4* had better outcomes associated with ibrutinib monotherapy. Specifically, patients with *MYD88* L265P *CXCR4* WHIM had lower response rates to single-agent ibrutinib compared to patients harboring the *MYD88* L265P *CXCR4* wild-type genotype, and progression free survival was inferior for previously treated patients with *MYD88* L265P *CXCR4* WHIM as compared to those with *MYD88* L265 *CXCR4* wild-type genotype (51, 53, 60, 61). Furthermore, although the addition of ibrutinib to rituximab resulted in a significant longer progression free survival regardless of the *MYD88* and *CXCR4* mutational status, major response rates were lower in patients with *CXCR4* WHIM (55). Interestingly, response outcome to ibrutinib therapy could be modulated by the subtype of *CXCR4* mutation. Ibrutinib-treated WM patients with nonsense *CXCR4* mutations showed fewer major responses and shorter progression-free survival compared with those wild-type for *CXCR4.* By contrast, frameshift *CXCR4* mutations were not associated with worse major response or progression free survival rates than those observed in WM patients with *CXCR4* wild-type genotype (62). Unlike *MYD88*, multiple *CXCR4* mutations can be present in an individual appearing with heterozygous character in different clones (63). Gustine et al. studied the impact of the *CXCR4* S338X (C1013G) nonsense variant, the most common *CXCR4* mutation identified, on ibrutinib response outcome in patients with WM. Ibrutinib-treated patients with a *CXCR4* S338X clonality ≥25% had lower rates of very good partial response, delayed major response attainment and a shorter median progression-free survival as compared to those with *CXCR4* S338X clonality <25% or a CXCR4 wild-type disease (64). These clinical data are consistent with the results of preclinical studies. Following stimulation with CXCL12, cultured WM cells engineered to express the *CXCR4* S338X mutation exhibited persistent activation of AKT and ERK and decreased apoptotic changes following ibrutinib treatment, compared to *CXCR4* wild-type cells (17). Moreover, WM cells harboring the *CXCR4* S338X mutation disseminated and proliferated more rapidly in the bone marrow and other organs of immunodeficient SCID mice, leading to disease progression and decreased survival (65) and demonstrated resistance *in vitro* to multiple drugs, including ibrutinib, the PI3K inhibitor idelalisib, and the mTOR inhibitor everolimus (17, 65). These studies also showed that resistance to ibrutinib mediated by mutated *CXCR4* can be reversed by use of the small-molecule CXCR4 antagonist plerixafor (AMD3100) (17) and that the CXCR4 antagonist ulocuplumab (BMS-936564/MDX-1338), a fully humanized IgG monoclonal antibody, displays anti-WM activity both *in vitro* and in WM-bearing mice, regardless of the *CXCR4* mutational status (65). Ulocuplumab is currently being evaluated in combination with ibrutinib in a phase 1/2 clinical trial for patients with relapsed or refractory (R/R) WM, who harbor a *CXCR4* mutation (NCT03225716). Mavorixafor (AMD-070), an orally active small-molecule allosteric antagonist of CXCR4, is also currently under evaluation in combination with ibrutinib in *MYD88* mutated WM patients harboring an additional *CXCR4* mutation (NCT04274738).

Although ibrutinib produces high response rates and durable remissions in WM, acquired mutations in BTK at the binding site of ibrutinib (C481), or in its downstream mediator phospholipase C γ2, have been identified in approximately half of WM patients who experienced disease progression on ibrutinib therapy (66). Multiple BTK mutations were observed within individual patients with WM, particularly those with *CXCR4* mutations, and appeared to be primarily subclonal (66). An *in vitro* study by Chen et al. demonstrated that BTK C481S, i.e. the most common BTK mutation in WM patients, results in sustained ERK1/2-mediated survival signaling and ibrutinib resistance in *MYD88*-mutated WM and large B-cell lymphoma cells and confers protection against ibrutinib to neighboring *BTK* wild-type cancer cells through cytokine release (67). More recently, a whole exome sequencing study demonstrated that acquired ibrutinib resistance in WM patients can be also associated with chromosome deletions in 6q and 8p that contain regulators of BTK, MYD88/NF-κB and apoptotic signaling, as well as with mutations in ubiquitin ligases, innate immune signaling, and TLR/MYD88 pathway regulators (68). As stated above, ibrutinib has a favorable toxicity profile but has multiple adverse effects, conceivably due to its low selectivity towards the target protein BTK (48). The quest for more selective BTK inhibitors has led to the discovery of the so called “second-generation” BTK inhibitors. Among these, zanubrutinib and acalabrutinib are orally active covalent inhibitors targeting C481 in BTK recently approved by the FDA and the EMA for the treatment of a group of B-cell malignancies. Preclinically, both compounds have shown to be more specific inhibitors of BTK than ibrutinib, with little impact on ibrutinib off-target kinases (69, 70). Zanubrutinib was found to be well tolerated and highly active in a phase I/II study in patients with TN and R/R WM (71). A subsequent randomized phase III study in TN and R/R WM patients harboring the *MYD88* L265P mutation evaluated zanubrutinib monotherapy in a head-to-head comparison with single agent ibrutinib (ASPEN trial). Although not statistically significant, zanubrutinib treatment was associated with a higher frequency of very good partial responses, than ibrutinib treatment. In addition, a lower rate of important adverse events, in particular cardiovascular events, was observed among patients who received zanubrutinib (72). The ASPEN study had also a second cohort of zanubrutinib-treated patients with *MYD88* wild-type WM (73); response rates in this cohort were considerably higher than those recorded by Treon et al. in ibrutinib-treated patients with R/R WM which were wild-type for both *MYD88* and *CXCR4* (51, 59). In 2020 the EMA has accepted a marketing authorization application for zanubrutinib for the treatment of patients with WM who have received at least one prior therapy or as frontline treatment for patients who are ineligible for chemoimmunotherapy; in April 2021 the FDA has accepted a supplemental new drug application for zanubrutinib for the treatment of adults with WM (74).

Notably, the presence of *CXCR4* mutations seems to affect the clinical outcome in the treatment of WM of not only ibrutinib but also of zanubrutinib. Indeed, in the ASPEN trial, fewer very good partial responses occurred in *CXCR4* WHIM patients treated with zanubrutinib (72). Furthermore, in a phase II trial involving patients with R/R WM the rates of very good partial response, major response, and overall response to zanubrutinib were higher in *MYD88* L265P *CXCR4* wild-type patients than in those with *MYD88* L265P *CXCR4* WHIM genotype (75).

A phase II trial investigated the efficacy and safety of acalabrutinib monotherapy in patients with WM. Drug treatment achieved high response rates both among TN and R/R WM patients. In this study, *MYD88* mutational status was assessed in a limited subset of patients (n=50) who were genotyped; the overall response rate was higher among *MYD88* L265P patients (94%) than among *MYD88* wild-type patients (79%), and no very good partial responses were observed in the *MYD88* wild-type patient group. Acalabrutinib showed a toxicity profile similar to that of ibrutinib, but a lower rate of atrial fibrillation (76). Combination therapy and further trials are warranted to elucidate the role of acalabrutinib in WM treatment as well as to evaluate the impact of *MYD88* and *CXCR4* mutational status on clinical outcomes.

As stated above, BTK C481 mutations are commonly observed in WM patients who experienced progression on ibrutinib (66). C481 mutations have also been observed in acalabrutinib and zanubrutinib treated patients with chronic lymphocytic leukemia undergoing disease progression (77, 78). Unlike ibrutinib and second-generation BTK inhibitors, next-generation BTK inhibitors such as vecabrutinib (SNS-062), pirtobrutinib (LOXO-305), and MK1026 (ARQ-351), are reversible inhibitors which do not interact with the BTK C481 site and can therefore conceivably overcome resistance associated with mutations at this residue. Some of these agents are currently undergoing clinical trials in patients with B-cell lymphoproliferative disorders, including WM, and preliminary results are encouraging (79).

## Conclusions

LPL/WM is a low-grade lymphoid malignancy for which novel therapies have been developed over the last years. Despite this progress, LPL/WM remains incurable and disease recurrence is the rule. As for other B-cell derived neoplasms, clinicians are increasingly called to be aware of the mechanisms of action and of resistance to novel drugs now available in the therapeutic armamentarium of LPL/WM. This is particularly true for kinase inhibitors (KIs), which are gaining a growing place in the therapy of this disease. Mutations of the KIs’ target, coexistence of genetic alterations, overactivation of compensatory signaling pathways and other mechanisms have been now recognized as central in the development of neoplastic clones able to escape the cytotoxic effects of KIs. A deep knowledge of the mechanisms of drug resistance will likely facilitate the design of novel more effective combination therapies. Another important question is whether the features of the relapse (i.e. indolent *versus* more aggressive) could be influenced by the sequential use of the available drugs. However, a clear answer to this issue is still awaited.

In conclusion, if it is predictable that LPL/WM will hardly be a B-cell malignancy for which definite complete responses could be attainable, it is likely that the future achievements of novel therapies will consist chiefly in prolonging at most the progression free and treatment free survival without affecting the quality of life of the patients.

## Author Contributions

FP, SM, and LQ conceived and design the paper. FP, VDP, and LQ read the relevant literature and wrote the paper. SM, GS, and LT provided data, insights and read the relevant literature. All authors contributed to the article and approved the submitted version.

## Conflict of Interest

The authors declare that the research was conducted in the absence of any commercial or financial relationships that could be construed as a potential conflict of interest.

## Publisher’s Note

All claims expressed in this article are solely those of the authors and do not necessarily represent those of their affiliated organizations, or those of the publisher, the editors and the reviewers. Any product that may be evaluated in this article, or claim that may be made by its manufacturer, is not guaranteed or endorsed by the publisher.
